# T cells, particularly activated CD4^+^ cells, maintain anti-CD20-mediated NK cell viability and antibody dependent cellular cytotoxicity

**DOI:** 10.1007/s00262-021-02976-7

**Published:** 2021-06-10

**Authors:** Zhaoming Wang, Michael S. Chimenti, Christopher Strouse, George J. Weiner

**Affiliations:** 1 Cancer Biology Graduate Program, Holden Comprehensive Cancer Center, Carver College of Medicine, The University of Iowa, Iowa City, IA USA; 2 Iowa Institute of Human Genetics, Carver College of Medicine, The University of Iowa, Iowa City, IA USA; 3 Department of Internal Medicine, Carver College of Medicine, The University of Iowa, Iowa City, IA USA

**Keywords:** NK cell, ADCC, Rituximab, T cell, Bispecific antibody

## Abstract

Anti-CD20 monoclonal antibody (mAb) therapy is a mainstay of therapy for B cell malignancies, however many patients fail to respond or eventually develop resistance. The current understanding of mechanisms responsible for this resistance is limited. When peripheral blood mononuclear cells of healthy donors were cultured with Raji cells for 7 days, rituximab (RTX) induced NK cell-mediated antibody-dependent cellular cytotoxicity (ADCC), enhanced NK cell viability and increased or maintained NK expression of CD56, CD16, CD57 and KIR. T cells, mainly CD4^+^, mediated these changes in a contact-dependent manner, with local T cell production of IL2 playing a central role. Similar findings were found when autologous B cells were used as target cells demonstrating the need for T cell help was not due to allogenic reaction. Results with other anti-CD20 and anti-EGFR antibodies were consistent. Small numbers of T cells activated by anti-CD3/CD28 beads or bispecific antibody enhanced RTX-mediated NK cell ADCC, viability and phenotypical changes. Pathway analysis of bulk NK cell mRNA sequencing after activation by RTX with and without T cells was consistent with T cells maintaining the viability of the activated NK cells. These findings suggest T cell help, mediated in large part by local production of IL2, contributes to NK cell ADCC and viability, and that activating T cells in the tumor microenvironment, such as through the use of anti-CD3 based bispecific antibodies, could enhance the efficacy of anti-CD20 and other mAb therapies where NK-mediated ADCC is a primary mechanism of action.

**Supplementary Information:**

The online version contains supplementary material available at 10.1007/s00262-021-02976-7.

## Introduction

Therapeutic anti-tumor monoclonal antibody (mAb) became a standard and important component of cancer therapy starting with rituximab (RTX), an anti-CD20 mAb. However, some B cell malignancies fail to respond or recur following anti-CD20-based therapy. Understanding the mechanism of action by which RTX mediates its anti-tumor efficacy is critical for improving its efficacy. Multiple mechanisms likely contribute to the therapeutic effects of RTX, including antibody-dependent cellular cytotoxicity (ADCC), complement dependent cytotoxicity, direct apoptosis and phagocytosis[1–5]. NK cell-mediated ADCC is believed to play an important role in mediating the anti-tumor activity of RTX in humans[1–5]. Human NK cells are typically defined as CD3^−^CD56^+^ lymphocytes and are divided into two subsets: CD56^dim^ and CD56^bright^. CD56^dim^ NK cells make up the majority (~ 90%) of circulating NK cells and express high levels of CD16[6]. They are considered to be the main effectors for ADCC. A smaller subset of NK cells is CD56^bright^ and expresses lower levels of CD16. The accepted paradigm is that CD56^dim^ NK are more mature and evolve from CD56^bright^ NK[7]. During this differentiation from CD56^bright^ to CD56^dim^, NK cells decrease the expression of c-kit, CD127 and CD62L while increasing the expression of CD57, KIRs and CD16[8]. Functionally, CD56^dim^ NK cells gain more cytotoxicity and lose proliferative potential[6, 8, 9]. The short-term effects of RTX on NK cells using 4 to 20-h assays have been extensively studied. However, the median half-life of RTX in non-Hodgkin lymphoma patients is 76 h and 206 h, respectively, after the first and fourth infusion[10]. The long-term effect of RTX on NK cells remains an important but understudied question. Here, we evaluate the long-term effect of RTX, and other anti-cancer monoclonal antibodies, on NK cell function and phenotypical changes, and the effect of T cells on NK response.

## Methods

### Samples and reagents

Peripheral blood mononuclear cells (PBMC) were isolated from healthy donors (DeGowin Blood Center) using gradient centrifugation. Following informed consent, Holden Comprehensive Cancer Center patients receiving weekly single agent RTX treatment provided peripheral blood collected before the 1^st^, 2^nd^, and 4^th^ RTX infusion for analysis. The use of human samples was approved by the Institutional Review Board. Raji B cell lymphoma cells were obtained from ATCC. SQ20B cells were provided by Andrean Simons-Burnett at the University of Iowa. RTX, obinutuzumab (OBZ), cetuximab (CTX) and trastuzumab (TRA) were from University of Iowa Hospitals & Clinics. 1DT3D was developed as previously reported[11]. CellTrace CFSE, CellTracker Red CMTPX dye, Dynabeads Human T-Activator anti-CD3/28 and CountBright Absolute counting beads were from Thermo Fisher.

### Cell coculture

For most analyses, 1 million PBMC were co-cultured with 0.2 million Raji cells after addition of RTX, OBZ or TRA at a concentration of 1ug/ml in a total volume of 200ul in round bottom 96-well plates (Corning). For the Transwell assay, the 96-well Transwell system (1um pore size, Corning) was used to separate T cells from T cell-depleted PBMCs. RMPI was supplemented with 10% FBS, 100 U / ug/ml Penicillin / Streptomycin, 2 mM L-Glutamine and 50 mM β-mercaptoethanol to make complete medium. α-IL2 (5334, R&D), α-IFNγ (B27, BioLegend), α-CD54 (HCD54, BioLegend), α-FGFR1(133,111, R&D) mAb, α-CD16 polyclonal Ab (R&D) or recombinant human IL2 (PeproTech) was added to the coculture in specific experimental settings. Complete medium including Abs or IL2 was refreshed every 2 days**.**

### Cell purification and depletion

Cell isolation kits (Miltenyi Biotec) were used to isolate NK cells, B cells, CD3^+^, CD4^+^ and CD8^+^ T cells by negative selection. Microbeads (Miltenyi Biotec) were used to deplete CD14^+^ monocytes, CD19^+^ B cells, CD3^+^, CD4^+^ or CD8^+^ T cells by LD columns. CD56^dim^ NK were separated from CD56^bright^ NK cells by flow sorting.

### Flow cytometry

Staining antibodies (BioLegend) included anti-human CD3 (HIT3a), CD56 (HCD56), CD14 (HCD14), CD19 (HIB19), CD16 (3G8), CD57 (HNK-1), KIR2DL1/S1/S3/S5 (HP-MA4), CD69 (FN50), CD25 (M-A251), CD54 (HCD54). Cells were first washed with PBS and stained with Zombie Aqua Fixable viability dye (BioLegend), followed by incubation with staining antibodies for 15 min at 4 °C. Samples were fixed in 2% formaldehyde and read by flow cytometry within 24 h. Data were analyzed using FlowJo v10.7 (FlowJo LLC).

### Proliferation assay

Cells were washed with PBS and stained with 5uM CFSE per manufacturer recommendations. Proliferation was quantified by determining the percent of cells with CFSE dilution. The absolute cell count was calculated using flow counting beads.

### mRNA sequencing

RNA was extracted from isolated NK cells using RNeasy Mini Kit (QIAGEN). Transcription profiling using RNA-Seq was performed using manufacturer recommended protocols. Briefly, 500 ng of DNase I-treated total RNA was used to enrich for poly-A containing transcripts using oligo(dT) primers bound to beads. The enriched RNA pool was fragmented, converted to cDNA and ligated to sequencing adaptors containing indexes using the Illumina TruSeq stranded mRNA sample preparation kit. The molar concentrations of the indexed libraries were measured using the 2100 Agilent Bioanalyzer and combined equally into pools for sequencing. The concentration of the pools was measured using the Illumina Library Quantification Kit (KAPA Biosystems) and sequenced on the Illumina NovaSeq 6000 genome sequencer using 100 bp paired end SBS chemistry.

### Bioinformatic analysis

Sequencing reads were processed with ‘*bcbio-nextgen*’ (v1.2.2) for QC, alignment, and read quantitation. The *bcbio-nextgen* pipeline was used to run ‘*MultiQC*’[12] and ‘*qualimap’* (2.2.2)[13]. Read were aligned against the GRCh37 reference genome using ‘*hisat2*’ aligner (2.2.0)[14] and concurrently quantified reads to the transcriptome using the alignment-free tool ‘*salmon*’ (1.1.0) aligner[15]. Transcript expression estimates were summarized from ‘*salmon’* output to the gene level using ‘*tximport’* (1.12.3) in R. Genes with fewer than five gene-level counts across all samples were excluded from downstream analysis. Differential gene expression analysis was conducted using *DESeq2* (1.24.0)[16]. An FDR of 1% was set as a cutoff for differential expression genes (DEGs). Principal component analysis plots were created with ‘*pcaExplorer’* [17]. The DEG data were analyzed using Advaita Bio’s iPathwayGuide[18–21].

### Statistics

Student’s t test was used to compare two independent groups. One-way or two-way ANOVA was used for multiple comparisons between different groups. Data were presented as mean ± SEM. All the analysis was performed in GraphPad Prism8. *p<*0.05 was considered to be statistically significant. * indicates *p* < 0.05, ** *p* < 0.01, *** *p* < 0.001, **** *p* < 0.0001, ns: not significant.

## Results

### Long-term exposure to RTX impacts on NK cell function and phenotype

**Fig. 1:**
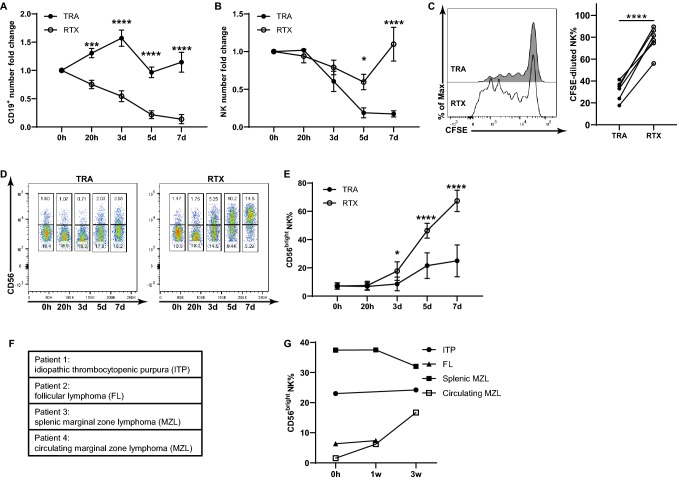
Long-term exposure to RTX impacts NK cell function and phenotype. PBMC were cocultured with Raji cells and RTX or TRA. NK cell function and phenotype were examined at different time points. **a** CD19^+^ target cells are progressively eliminated over time in response to RTX. **b** RTX maintains the number of NK cells within PBMCs. **c** RTX induces proliferation of NK cells within PBMC at 7 days as determined by CFSE dilution. **d, e** RTX progressively increases the percent of CD56^bright^ NK cells beginning at day 3. n = 6. Cell counts at 0 h were used to normalize cell numbers. **f** Patient 4 has circulating tumor cells, while the others have no detectable circulating tumor cells. **g** The fraction of CD56^bright^ NK cells increased following RTX in the patient with circulating malignant cells but not in patients without circulating malignant cells

**Fig. 2:**
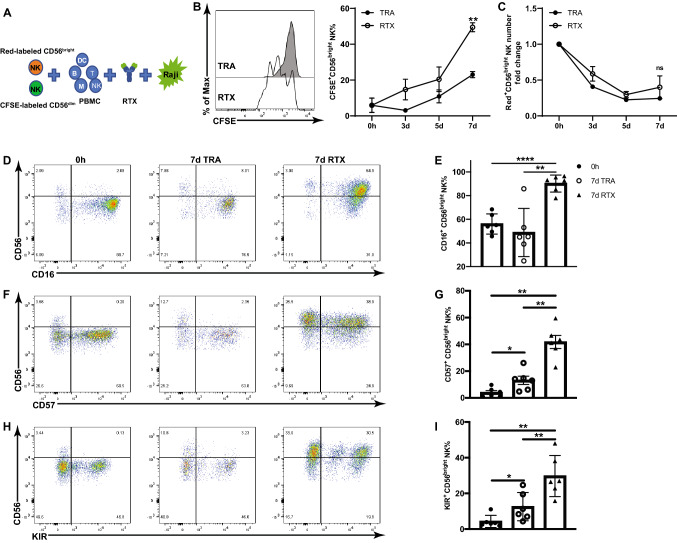
CD56^dim^ NK cells transit into CD56^bright^ NK cells in response to long-term RTX activation. **a** Isolated CD56^dim^ and CD56^bright^ NK cells were stained with CFSE and CellTracker Red, respectively. CFSE-labeled CD56^dim^ and Red-labeled CD56^bright^ NK were added to autologous PBMC and cocultured with Raji cells and RTX or TRA. **b** As illustrated by a day 7 histogram gated on CFSE-labeled CD56^dim^ NK cells, CFSE-labeled CD56^dim^ NK cells proliferate and adopt a CD56^bright^ phenotype in the RTX group. n = 3. **c** The number of CellTracker Red labeled-CD56^bright^ NK cells does not increase in response to RTX. n = 3. **d–i** CD16, CD57 and KIR were largely expressed by resting CD56^dim^ NK cells. RTX-induced CD56^bright^ NK cells express high levels of CD16, CD57 and KIR. n = 6. Cell counts at 0 h were used to normalize cell numbers

Initial studies evaluated the impact of RTX on ADCC as well as the number and phenotype of NK cells in longer term cultures of PBMCs and Raji cells. The elimination of target B cells by RTX over time suggested ongoing ADCC (Fig. 1a). NK cells in these cultures persisted and proliferated as demonstrated by the maintenance in NK cell number (Fig. 1b) and CFSE dilution (Fig. 1c). After 5–7 days, NK cells within PBMCs cultured with Raji cells and RTX shifted from CD56^dim^ to CD56^bright^ (Fig. 1d, e). This change was not seen when TRA was added instead of RTX. OBZ, an anti-CD20 mAb recognizing a different-oriented epitope from RTX, showed changes consistent with those seen with RTX (Fig. S1a, b). Change in NK cell phenotype in vivo was determined using peripheral blood samples from patients receiving weekly single agent RTX (Fig. 1f). The fraction of NK cells with the CD56^bright^ phenotype increased following RTX in the patient with circulating malignant cells but not in the patients without circulating malignant cells (Fig. 1g, Fig. S1c). Further studies were done to assess whether the shift in CD56^dim^ and CD56^bright^ NK cells derived from the differential expansion of the two subsets, or a shift of CD56^dim^ to CD56^bright^ cells. Sorted CD56^dim^ and CD56^bright^ NK cells were labeled with CFSE and CellTracker Red, respectively. Labeled CD56^dim^ and CD56^bright^ NK cells were added back to autologous PBMC and cultured with Raji cells and RTX (Fig. 2a). After 7 days, CFSE-labeled CD56^dim^ NK cells proliferated and displayed a CD56^bright^ immunophenotype (Fig. 2b), while CellTracker Red-labeled CD56^bright^ cells did not expand (Fig. 2c). These studies demonstrate CD56^dim^ NK cells within PBMCs proliferate and increase CD56 expression after longer-term culture with Raji cells and RTX. Additional maturation markers were assessed to better understand the differentiation status of the CD56^bright^ NK cells that emerge following longer-term culture with RTX. Resting CD56^bright^ NK cells express low levels of CD16 while resting CD56^dim^ NK cells express higher levels of CD16. The expression of CD16 by CD56^dim^ is known to be downregulated on NK cells in response to short-term RTX activation[22, 23]. However, expression of CD16 on NK cells recovered after 7 days with the majority of CD56^bright^ NK cells expressing CD16 (Fig. 2d, e). These cells also expressed CD57 and KIR (Fig. 2f–i) which, in the resting state, are expressed largely by CD56^dim^ NK cells. Less pronounced changes in NK cell phenotype and some control of target cell growth were seen in the TRA control group, likely due to allogeneic NK response to Raji cells. Together, this phenotypic data suggest that RTX-activated CD56^dim^ NK cells upregulate the expression of CD56, re-express high levels of CD16, and display other markers of mature NK cells.

### T cells are required for maintaining RTX-mediated NK cell responses

**Fig. 3:**
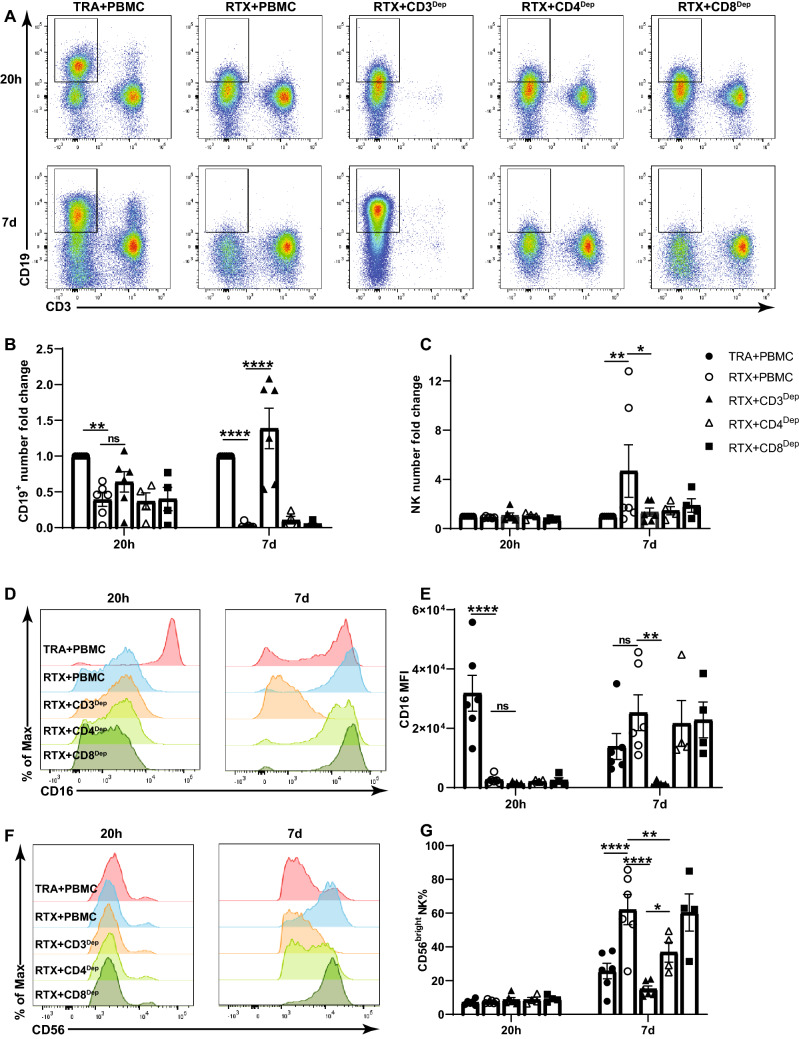
T cells are required for RTX-mediated NK cell cytotoxicity, viability, CD16 re-expression and CD56^dim^ to CD56^bright^ transition. Unfractionated PBMC or PBMC depleted of CD3^+^, CD4^+^, or CD8^+^ cells were cocultured with Raji cells and RTX or TRA. **a, b** CD19^+^ target cells are mostly eliminated by RTX on day 7. RTX-mediated elimination of CD19^+^ cells is inhibited by the depletion of CD3^+^ T cells. **c** The number of NK cells remaining in intact PBMCs is maintained by RTX but not by TRA. However, NK cell numbers are not maintained after CD3^+^ T cell depletion. **d, e** The expression of CD16 is downregulated on NK cells by RTX activation at 20 h and recovers on day 7. CD16 re-expression is not observed after CD3^+^ T cell depletion. **f, g** CD56^dim^ to CD56^bright^ NK transition is induced in unfractionated PBMC after 7 days. This transition is inhibited by depletion of CD3^+^ or CD4^+^ cells but not after depletion of CD8^+^ cells. Cell counts in the TRA + PBMC group were used to normalize cell numbers. n = 4–6. Dep: depleted

**Fig. 4:**
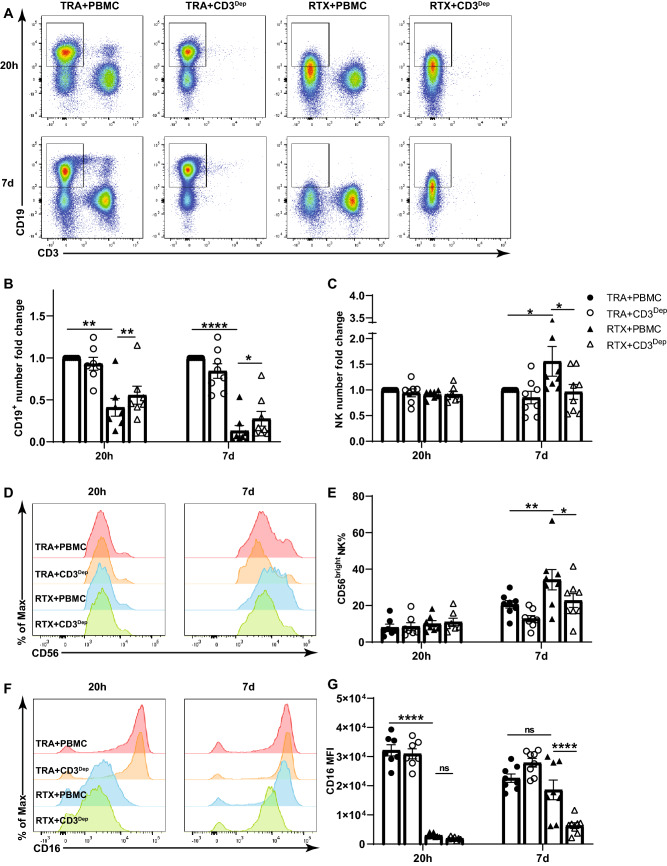
T cells are required for RTX-mediated NK cell responses in the autologous system. Unfractionated PBMC or PBMC depleted of CD3^+^ cells were cocultured with enriched numbers of autologous B cells and TRA or RTX. RTX-mediated elimination of CD19^+^ autologous B cells (**a, b**), the number of NK cells (**c**), CD56^dim^ to CD56^bright^ transition (**d, e**) and CD16 re-expression (**f, g**) on NK cells is suppressed by the depletion of CD3^+^ T cells after 7 days. Cell counts in the TRA + PBMC group were used to normalize cell numbers. n = 7–8. Dep: depleted

NK cells were isolated from PBMC and cocultured with RTX and Raji cells for 7 days. In contrast to what was observed with NK cells in unfractionated PBMCs, RTX failed to induce CD56^dim^ to CD56^bright^ transition, CFSE dilution or CD16 re-expression by isolated NK cells (Fig. S2a–d). The number of NK cells remaining in the RTX group was higher than that in the TRA group but this difference was considerably less than was seen with unfractionated PBMCs (RTX to TRA NK ratio – 3.05 versus 9.42, Fig. S2e). The elimination of Raji cells by RTX was limited when isolated NK cells were used as effector cells (Fig. S2f). This suggested a cell population in PBMC was maintaining NK cell growth, viability, cytotoxicity, and phenotypic change. To identify the cellular component in PBMC supporting these changes, monocytes, B cells or T-cell subsets were depleted and remaining cells cocultured with RTX and Raji cells. Down-modulation of CD19 in response to RTX was seen within 20 h which is consistent with previous reports[24]. T cell depletion had limited impact on elimination of target cells at 20 h (Fig. 3a, b). However after 7 days, depletion of CD3^+^ T cells inhibited NK cell ADCC, viability and CD16 re-expression (Fig. 3a–e). The depletion of CD3^+^ or CD4^+^ T cells significantly suppressed the CD56^dim^ to CD56^bright^ NK transition after 7 days (Fig. 3f, g). Suppression of the CD56^dim^ to CD56^bright^ NK transition was most pronounced after CD3^+^ depletion, but was also seen with CD4^+^ depletion, suggesting CD4^+^T cells are primarily responsible for supporting CD56^dim^ to CD56^bright^ NK transition but that CD8^+^ T cells can contribute to this process. The expression of activation markers on NK cells including CD25 and CD69 was not altered by the depletion of CD3^+^ T cells (Fig. S3a, b). Depletion of B cells and monocytes had minimal impact on RTX-mediated NK cell cytotoxicity, viability, or phenotypical changes after 7 days (Fig. S4). To further assess the role of T cells in RTX-mediated ADCC, isolated NK cells were cocultured with RTX and Raji cells, and autologous CD3^+^, CD4^+^ or CD8^+^ T cells were added back before culturing for 7 days. RTX-mediated NK ADCC was enhanced and NK cell numbers were higher when CD3^+^ or CD4^+^ T cells were added back (Fig. S5a–c). CD56^dim^ to CD56^bright^ transition was not induced in isolated NK cells unless CD3^+^ T cells, CD4^+^ or CD8^+^ T cells were added. CD3^+^ and CD4^+^ T cells triggered more CD56^dim^ to CD56^bright^ NK transition than did CD8^+^ T cells (Fig. S5d, e). CD16 recovery was only seen with CD3^+^ or CD4^+^ T cells (Fig. S5f, g). Taken together, these data demonstrate that T cells, largely CD4^+^ cells, are required to maintain NK cell ADCC, viability, and phenotypical changes. It is possible an allogeneic reaction between T cells and Raji cells contributed to changes in the NK cell responses. To assess this possibility, RTX was added to PBMCs enriched for autologous B cells that served as target cells for RTX and cultured for 7 days. Results in this fully autologous system were similar to those seen with Raji as target cells. RTX-mediated NK elimination of autologous B cells, NK viability, CD56^dim^ to CD56^bright^ transition and CD16 re-expression were suppressed by the depletion of CD3^+^ T cells (Fig. 4). T cell depletion did not impact NK cell activation (Fig. S3c, d).

### IL2 in the immunological synapse contributes to the impact of T cells on NK cell function.

**Fig. 5:**
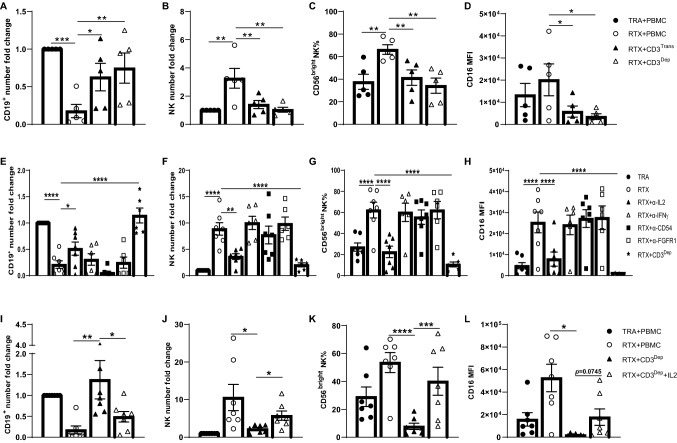
T cells impact RTX-mediated NK cell responses in a contact dependent manner. CD3^+^ T cells were depleted from PBMC and then added back to the lower Transwell chamber (with Raji and remaining PBMCs) or the upper chamber (separated from Raji and remaining PBMCs), then cultured with RTX or TRA for 7 days. **a–d** Elimination of CD19^+^ target cells, the number of NK cells, CD56^dim^ to CD56^bright^ NK transition and CD16 recovery by RTX activation is suppressed by the physical separation of T cells (CD3^Trans^) from the remainder of the PBMCs. n = 5. Unfractionated PBMCs were cocultured with Raji cells and RTX or TRA for 7 days. a-IL2, a-IFNg, a-CD54, or a-FGFR1 mAb (10ug/ml) was added to the coculture. **e–h** On day 7, IL2 neutralization suppressed RTX-mediated CD19^+^ target cell elimination, NK viability, CD56^dim^ to CD56^bright^ NK transition and CD16 re-expression by NK cells. n = 6–7. Unfractionated PBMC or PBMC depleted of CD3^+^ T cells were cocultured with Raji cells and RTX or TRA for 7 days. Recombinant IL2 (20 ng/ml) was added to the coculture. **i–l** On day 7, IL2 supplementation increased RTX-mediated cytotoxicity, viability, CD56^dim^ to CD56^bright^ transition and CD16 re-expression of NK cells in T cell-depleted PBMCs. n = 7. Cell counts in the TRA group were used to normalize cell numbers. Dep: depleted

A Transwell system was used to investigate whether the impact of T cells on RTX-activated NK cells was contact dependent. When CD3^+^ T cells were physically separated from NK cells, NK ADCC, viability, CD56^dim^ to CD56^bright^ transition and CD16 recovery was significantly reduced after 7 days (Fig. 5a–d). This suggests close contact between NK cells and T cells is needed to maintain the RTX-mediated NK cell response. Importantly, this does not exclude the possibility that soluble factors secreted by T cells impact on NK cells via the immunological synapse. T cells are known to interact with NK cells via a variety of ligand-receptor pairs including IL2–IL2R, IFNg-IFNgR, CD54-LFA1 and FGFR1–CD56[25–28]. To investigate the mechanism how T cells impact RTX-mediated NK cell responses, neutralization mAbs were used to block each of these pairs. Anti-IL2 significantly inhibited NK cell ADCC, viability, CD56^dim^ to CD56^bright^ transition and CD16-reexpression (Fig. 5e–h). Recombinant IL2 was sufficient to maintain NK cell response without the need for T cells (Fig. 5i–l). IL2 locally produced by T cells could have higher concentration in the immunological synapse, resulting in more profound effects on NK cells. Although IL2 alone is adequate to induce NK cell functional and phenotypical changes, other ligand-receptors pairs that require cell-to-cell contact may be involved as well. Therefore, T cells impact RTX-mediated NK cell response at least partially via IL2. The need for cell–cell contact suggests this interaction may be more robust in the immunological synapse.

### T cells maintain CTX-mediated NK cell responses

To assess whether the observations outlined above are limited to anti-CD20 mAb or B cells as target cells, similar studies were done evaluating changes of NK cells in response to CTX, an anti-EGFR mAb, and head and neck cancer cells. CTX induced NK cell CD56^dim^ to CD56^bright^ transition, maintained NK cell numbers and promoted CD16 recovery on NK cells (Fig. S6a–e) in a manner consistent with that seen with RTX. CTX-mediated effects on NK cells were dependent on the presence of CD3^+^ T cells and IL2 just as was seen with RTX (Fig. S6f–h). This indicates that T cells may be critical in maintaining the long-term NK cell response to a variety of mAb via IL2.

### T cell activation enhances RTX-mediated NK cell function.

**Fig. 6:**
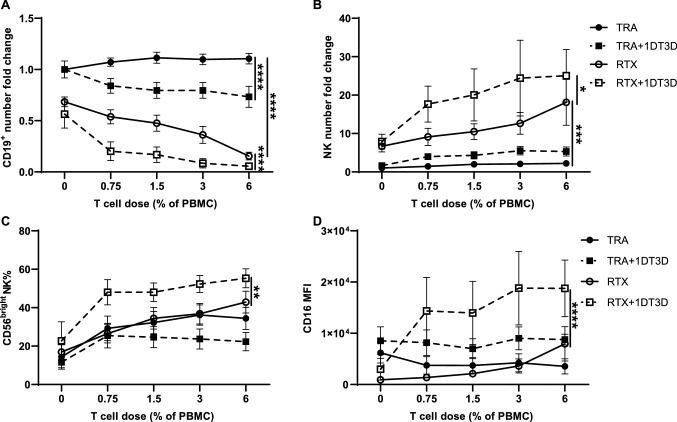
T cell activation enhances RTX-mediated NK cell responses. PBMC depleted of CD3^+^ T cells were cocultured with Raji cells and RTX or TRA for 7 days. Serial dilutions of either autologous resting or 1DT3D (0.5ug/ml) activated T cells (from 0.75 to 6% of the PBMC amount) were added to the coculture. **a–d** RTX-mediated NK cell cytotoxicity, viability, CD56^dim^ to CD56^bright^ transition and CD16 re-expression is T cell dose dependent and further enhanced by T cell activation. n = 5. Cell counts in the TRA group at 0% T cell dose were used to normalize cell numbers

Studies were done to determine whether activation of T cells enhances their ability to support NK cells. T cells were depleted from PBMC. Autologous resting T cells or T cells activated by anti-CD3/CD28 beads were added back in various concentrations. Activation of T cells enhanced NK cell responses particularly at lower doses of T cells (Fig. S7). Similar results were found following addition of a bispecific anti-HLA-DR/anti-CD3 monoclonal antibody developed in our laboratory[11] designated IDT3D (Fig. 6). 1DT3D at low concentrations enhanced NK cell ADCC, viability, CD56^dim^ to CD56^bright^ transition, and CD16 recovery in the presence of small numbers of T cells, in some cases less than 1% (Fig. 6). This suggests activation by bispecific anti-CD3 antibodies of small numbers of T cells in the tumor microenvironment could enhance RTX-mediated NK cell responses.

### The effects of T cells on RTX-activated NK cell transcriptomics

**Fig. 7:**
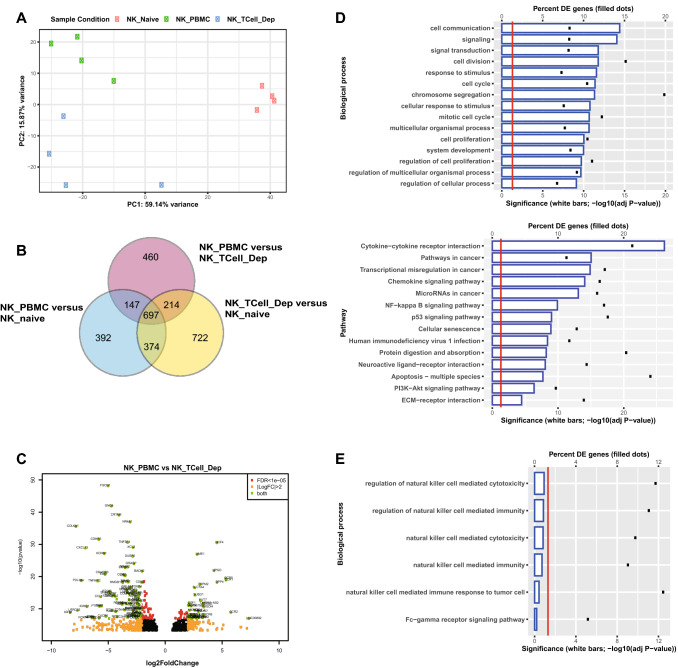
T cells impact RTX-mediated NK cell transcriptomics. **a** The transcriptomics of NK cells isolated from different experimental conditions: NK_naive, NK_PBMC and NK_TCell_Dep were easily separated by PCA. **b** Summary of DEGs from three conditions. **c** NK_PBMC versus NK_TCell_Dep volcano plot of DEG. **d** DEGs of NK_PBMC versus NK_TCell_Dep are mostly enriched in biological processes associated with cell communication and cell proliferation as determined by impact analysis in iPathwayGuide. Using this analysis, the top pathway enriched by DEGs of NK_PBMC versus NK_TCell_Dep is the cytokine – cytokine receptor interaction. **e** Depletion of T cells does not impact the biological pathways involved in NK cell cytotoxicity and Fcg receptor signaling at the transcriptional level

Bulk NK cell mRNA sequencing was used to evaluate the effects of T cells on the RTX-mediated NK cell response at the transcriptional level. PBMCs (unfractionated and after T cell depletion) were cultured for 7 days with RTX and Raji cells. NK cells were then isolated from three experimental conditions: (1) 0 h, resting PBMC (NK_naive), (2) intact PBMCs (NK_PBMC), (3) T cell-depleted PBMC (NK_TCell_Dep). Transcriptomics of NK cells from the three groups were well distinguished from each other by principal component analysis (Fig. 7a), indicating they were transcriptionally different. A prime focus for analysis was on how T cells impact on RTX-mediated NK cell transcriptomics (Fig. 7b, c). The top biological processes enriched by DEGs between the NK_PBMC and NK_TCell_Dep samples included cell communication, signaling and cell division, suggesting the importance of T-NK cell interaction in NK cell proliferation. Depletion of T cells also altered the “cytokine – cytokine receptor interaction” pathway (Fig. 7d), consistent with the finding that IL2 is playing an important role. The depletion of T cells did not have a significant impact on the Fcg receptor signaling or the NK cell cytotoxicity suggesting T cells have minimal direct impact on RTX-mediated NK cell activation (Fig. 7e). This analysis further supports the experimental findings that T cells impact on RTX-mediated NK cell response mainly by enhancing NK cell viability and proliferation, not by enhancing the cytotoxic potential of the NK cells.

## Discussion

The demonstration over 20 years ago that a mAb that targets a malignant cell, RTX, has significant and prolonged clinical anti-cancer activity has shifted the paradigm for treatment of B cell malignancies and other cancers. mAbs are now a standard component of treatment regimens for multiple cancers. Despite the remarkable success of anti-tumor mAbs, resistance and relapse remain common. RTX and other mAbs have remarkably long half-lives as drugs, with therapeutic levels being distributed in both the intravascular and extravascular compartments for weeks or even months during a course of therapy. The possible vaccinal effect of RTX suggests RTX impacts on the interaction between T cells and NK cells[29, 30]. This led us to explore the role of T cells in the long-term NK cell response to RTX. The overall goal of these studies was to enhance our understanding of the crosstalk between innate and adaptive immunity in the context of anti-tumor antibodies that could lead to improved mAb-based therapeutics. Proliferation of NK cells in response to anti-CD20 mAb has been previously reported in CLL samples when PBMCs containing NK cells, target cells and mAb are present together in the peripheral blood[31]. The results presented here that mAb can induce proliferation of NK cells are consistent with these findings. Previous studies reported that CD56^dim^ NK cells increase CD56 expression in response to various stimuli including the Bacillus Calmette-Guerin vaccine, engineered antigen presentation cells, and cytokines[32–34]. Here we found long-term activation of NK cells by mAb-coated target cells also induced CD56 upregulation. Furthermore, these transited CD56^bright^ NK cells also re-express CD16, express other maturation markers, and effectively mediate ADCC. The NK cell ability to mediate high levels of ADCC, viability, CD56^dim^ to CD56^bright^ transition, and re-expression of CD16 induced by RTX were seen with unfractionated PBMC but not with isolated NK cells, suggesting the interaction with other cell types plays a central role. Both CD4^+^ and CD8^+^ T cells contributed to this effect, with CD4^+^ playing a more prominent role. The role of T cells in anti-CD20 efficacy has been evaluated in mouse models with a focus on eventual development of an anti-lymphoma T cell response[35]. To our knowledge, our demonstration that T cells, particularly CD4^+^ cells, contribute to maintaining the viability of NK cells, thereby enhancing their ability to mediate ADCC, is a novel finding. Studies at the transcriptional level demonstrated T cells impact on differential expression of NK cell genes involved in NK cell viability and proliferation, with less of an impact on genes involved in NK cell activation or cytotoxicity. This finding is consistent with experimental findings that T cells impact on RTX-induced viability and proliferation of NK cells but not expression of activation markers. Together, these data suggest T cells mainly support NK cell viability to maintain ADCC as opposed to enhancing NK activation. Results from the Transwell and ligand-receptor blocking assays indicates T cells, largely via IL2 in the immunological synapse, support RTX-mediated NK cell function and phenotypical changes. This finding is consistent with studies of vaccination and infection where NK cell activation has been found to be dependent on T cell-derived IL2[36, 37]. Use of IL2 to enhance the efficacy of RTX and other mAb therapy is not a new concept. Clinical trials evaluating the combination of IL2 and RTX failed to benefit patients perhaps due in part to IL2 impact on regulatory T cells, which suppress NK function[38, 39]. The kinetics and toxicity of exogenously administered IL2 would be expected to be very different than that of IL2 produced in the tumor microenvironment by resident T cells. It is therefore possible inducing local production of IL2 by T cells in the tumor microenvironment will have a therapeutically important impact on NK cell mediated ADCC even though exogenously administered IL2 did not. Changes in RTX-mediated NK cells were dependent on T cell dose. Furthermore, activated T cells were more effective than resting T cells at providing T cell help to NK cells. This suggests one potential mechanism of resistance to mAb therapy is lack of an adequate number of intratumoral T cells to provide T cell help that maintains the ability of NK cells to mediate ADCC. The finding that intratumor T cell infiltration correlates with better prognosis in lymphoma patients who have received RTX-containing therapy[40–42] is consistent, although obviously does not prove, this hypothesis. A number of approaches could be used to activate intratumoral T cells with the goal of enhancing T cell help provided to NK cells. One such approach would be to combine standard mAb therapy with bispecific antibodies that can activate intratumoral T cells. Such combinations have been proposed before[43], but not based on the mechanism described here. Understanding this mechanism could impact on the design of such a regimen since the role of the bispecific antibody would not only be to induce T cell mediated killing of the target cell directly, but also to provide enough T cell help to support the NK cells that mediate ADCC. Intermittent dosing of lower dose bispecific antibody on a schedule similar to that for RTX or other mAb would help achieve this goal. Such an approach could obviate the need for continuous infusion and reduce toxicity associated with the cytokine storm seen with current approaches to bispecific antibody therapy[44]. While most of the studies reported here were done using RTX, consistent results were found with other mAb, such as CTX, that also appear to mediate much of their anti-tumor activity by ADCC suggesting this finding could have implications beyond anti-CD20 mAb therapy. Consideration was given to assessing the role of T cells in enhancing NK-cell mediated ADCC in murine models. Unfortunately, in vitro studies using mouse splenocytes and syngeneic tumor cells indicated that mouse NK cell phenotypic change, ADCC and viability is not maintained over long-term coculture in mice as it is in humans. In contrast to human NK, mouse NK cells have different FcgR binding patterns and don’t express CD56. Study of this particular mechanism in vivo in mouse models would be of limited clinical relevance because of these and other differences in the mechanisms of action by which anti-cancer mAbs mediate anti-tumor efficacy in mice and humans[1–5]. Additional studies in the human system, currently ongoing, are evaluating the phenotype of intratumoral NK cells in biopsies obtained before and after mAb therapy are exploring whether the in vitro findings described here also take place clinically. In addition, ongoing studies are evaluating the combined effects of other monoclonal antibodies and bispecific antibodies and the duration of such therapy required for T cell activation and its resulting support NK function. In conclusion, T cells, particularly activated CD4^+^ cells, maintain NK cell viability and the ability to mediate ADCC, and promote mAb-induced phenotypic change via local IL2 production after long term in vitro culture. These findings suggest maintaining intratumoral T cell activation, such as that mediated by CD3-based bispecific antibodies, could enhance NK cell viability and ADCC thereby improving the efficacy of anti-CD20, and other mAb therapies where NK-mediated ADCC is a primary mechanism of action.

## Supplementary Information

Below is the link to the electronic supplementary material.

Supplementary file1 (PDF 2071 kb)

## Data Availability

Sequencing data are deposited at NCBI GEO: GSE164086**.** All data are available on reasonable request.

## Code Availability

The code for bioinformatics analysis is available upon request.
