# Supplementary material for: T cells, particularly activated CD4+ cells, maintain anti-CD20-mediated NK cell viability and antibody dependent cellular cytotoxicity
Source: Cancer Immunol Immunother. 2021 Jun 10;71(2):237–49. doi: 10.1007/s00262-021-02976-7 (PMC8783893; doi:10.1007/s00262-021-02976-7)
Supplement: Supplementary file 1 — Supplementary file1 (PDF 2071 kb) [file 262_2021_2976_MOESM1_ESM.pdf]

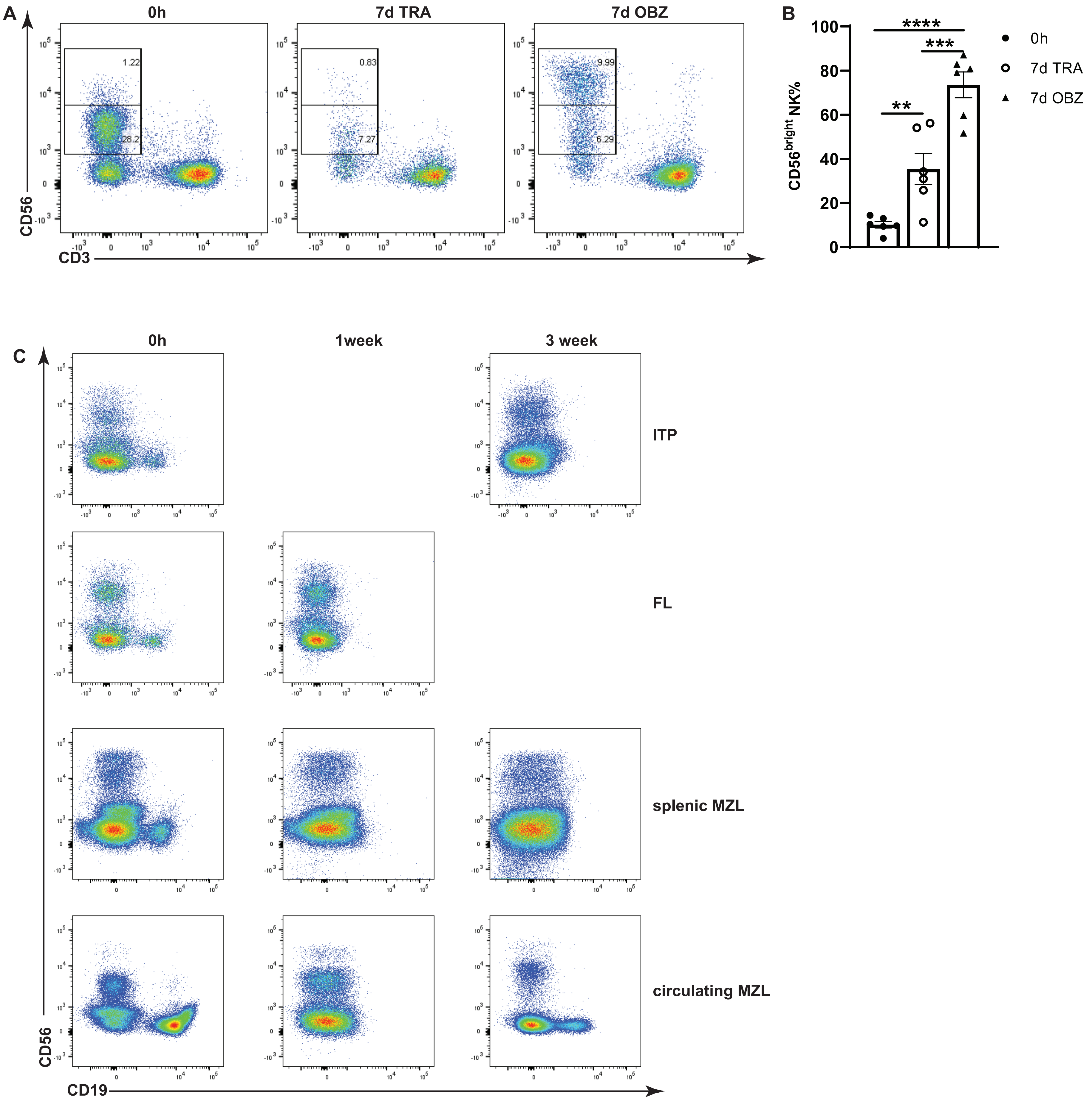

Supplementary Fig. 1 CD56^{dim} to CD56^{bright} NK cell phenotypical change in response to OBZ and in patients receiving RTX infusion. a, b PBMC were cocultured with Raji cells and OBZ or TRA for 7 days. OBZ induces enhanced expression of CD56 on NK cells. n=6. **c** Patients were treated by weekly single agent RTX infusion. CD19^{+} target cells were eliminated 1 week after RTX treatment, but re-emerged on week 3 in the patient with circulating tumors

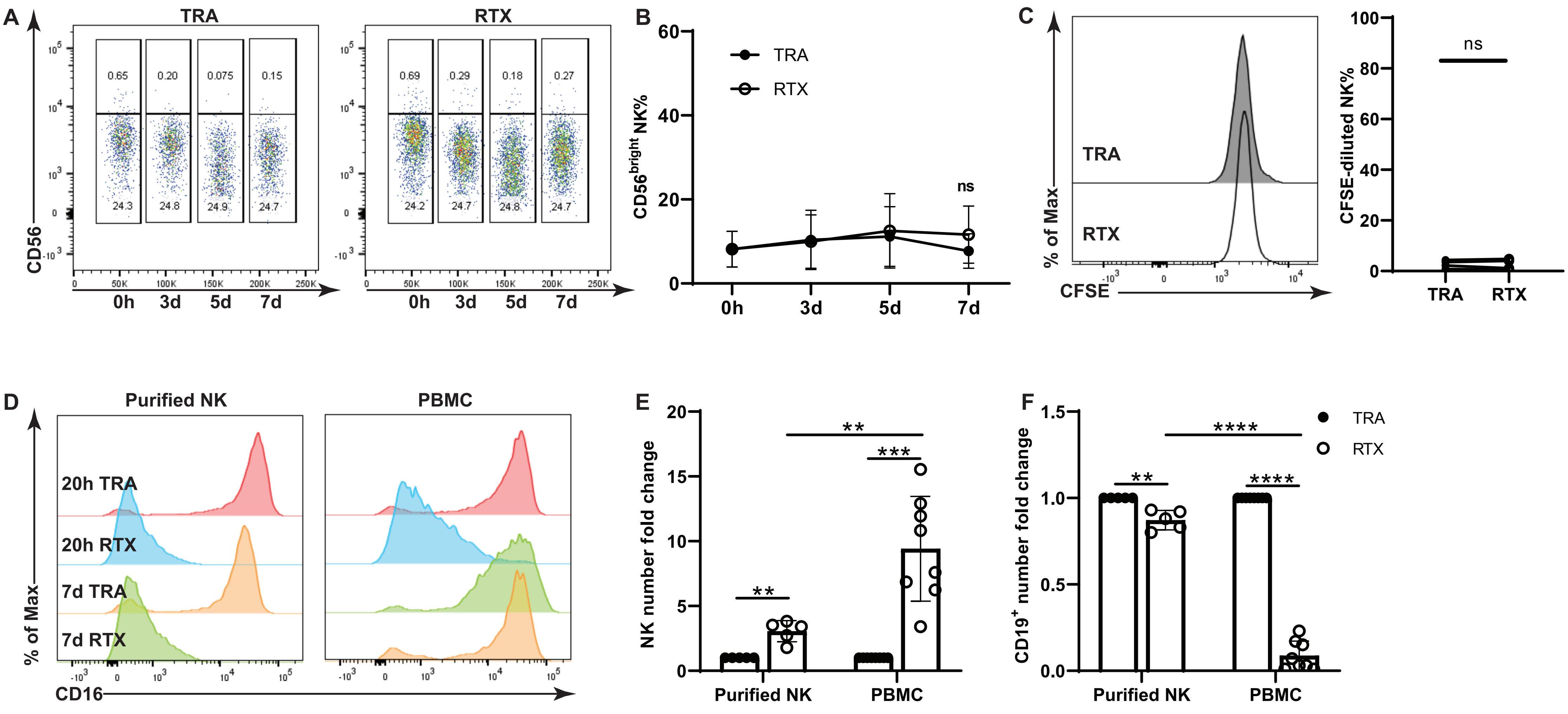

Supplementary Fig. 2 RTX fails to induce CD56^{dim} to CD56^{bright} transition and proliferation of isolated NK cells. Isolated NK cells or unfractionated PBMCs were cocultured with Raji cells and RTX or TRA for up to 7 days. **a, b** RTX has no impact on CD56 expression by isolated NK cells. n=4. **c** RTX fails to induce CFSE dilution by isolated NK cells at 7 days. n=5. **d** CD16 re-expression is seen with unfractionated PBMCs, but not with isolated PBMCs, after culture for 7 days with RTX. **e** The number of NK cells remaining after 7-day culture with RTX is greater with unfractionated PBMCs compared to isolated NK cells. **f** Elimination of CD19^{+} target cells after a 7-day culture is greater with unfractionated PBMCs compared to isolated NK cells. n=5-8. Cell counts in the TRA group were used to normalize cell numbers

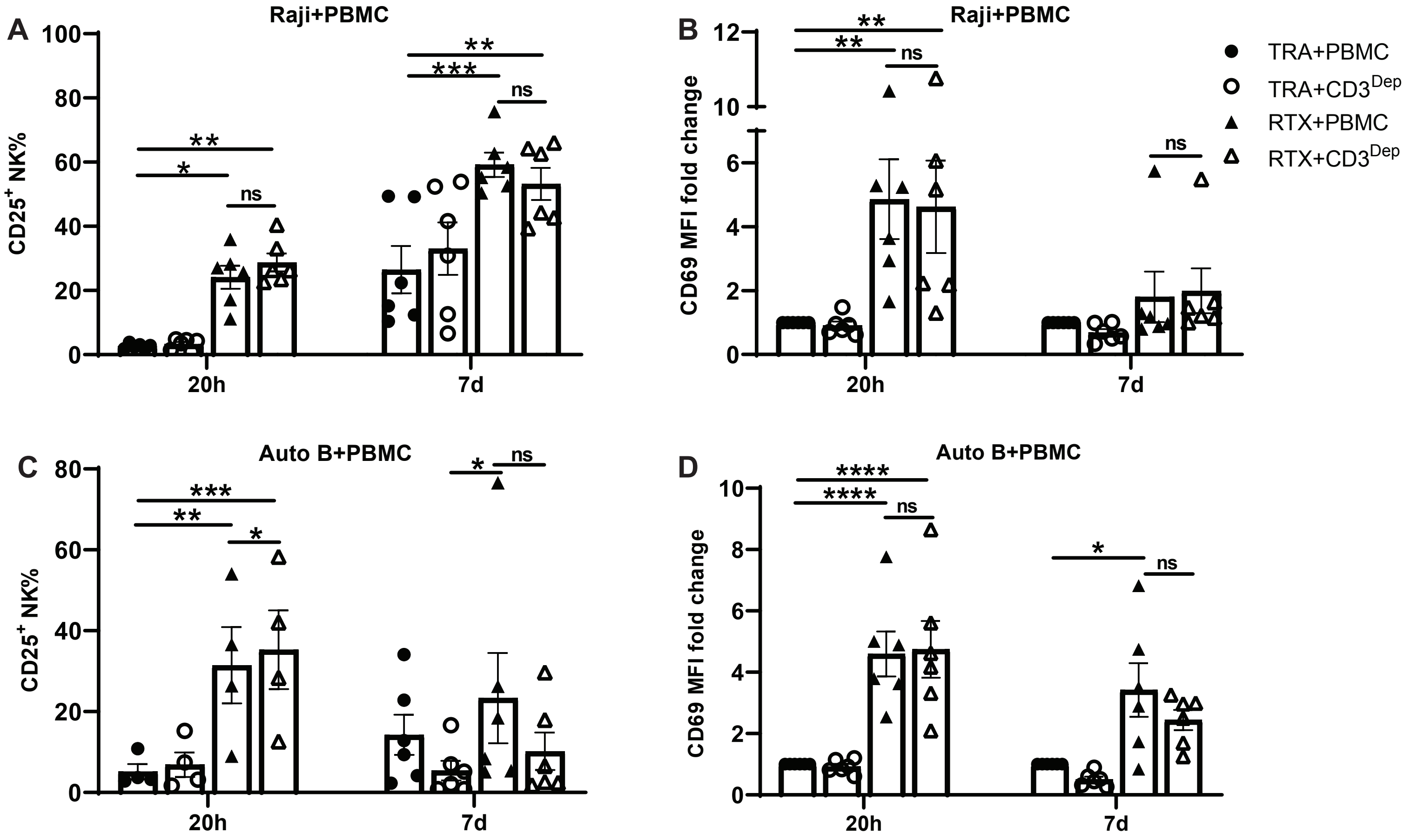

Supplementary Fig. 3 T cell depletion doesn't impact RTX-mediated NK cell activation. Unfractionated PBMC or PBMC depleted of $CD3^+$ cells were cocultured with Raji or autologous B cells and RTX or TRA for 7 days. Depletion of T cells does not alter RTX-activated NK expression of CD25 (**a, c**) or CD69 (**b, d**). n=4-6

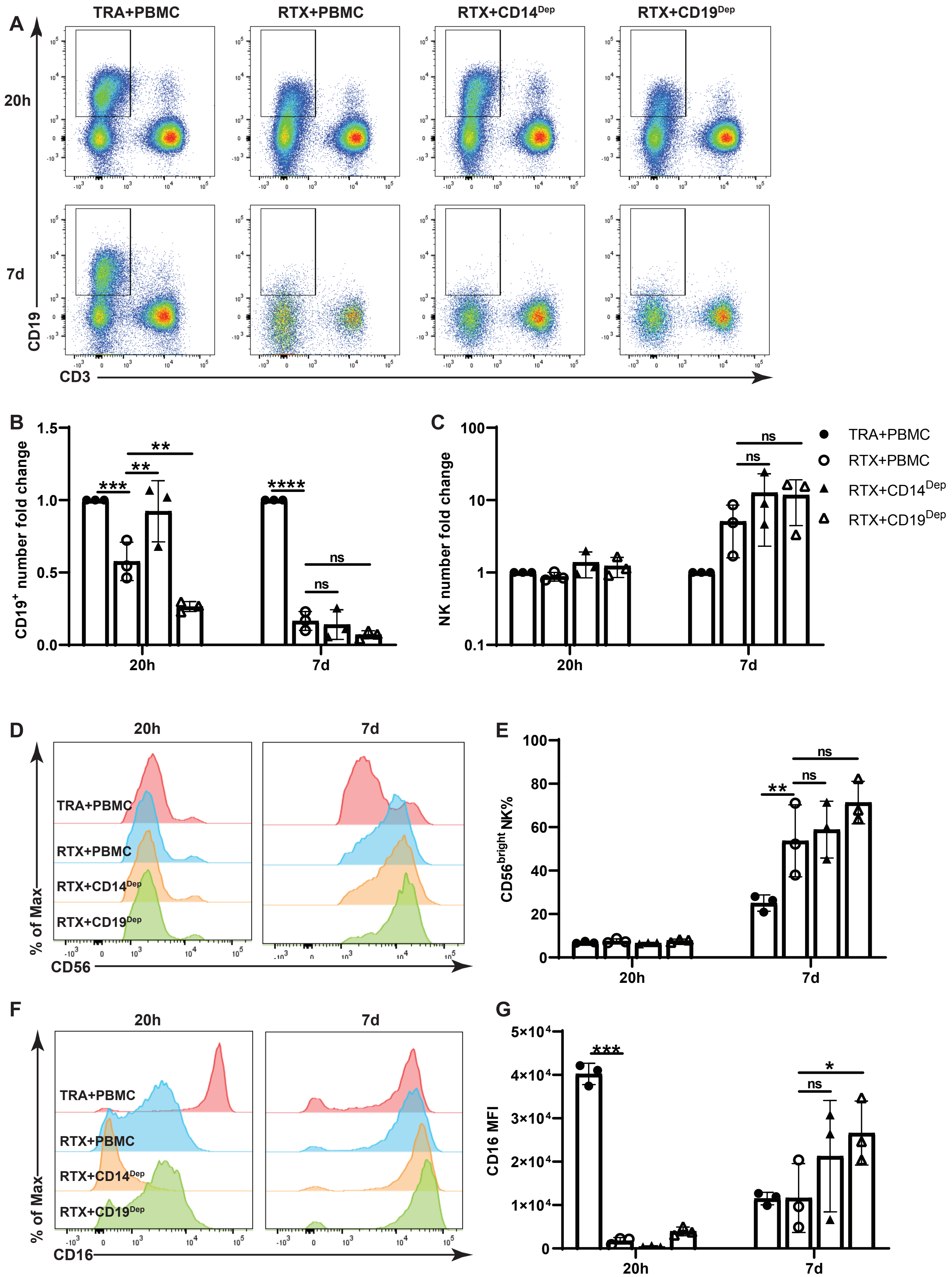

Supplementary Fig. 4 The depletion of CD14⁺ monocytes or CD19⁺ B cells does not suppress RTX-mediated NK cell responses. Unfractionated PBMC or PBMC depleted of CD14⁺ monocytes or CD19⁺ normal B cells were cocultured with Raji cells and RTX or TRA for 7 days and elimination of CD19⁺ target cells determined by flow cytometry. **a, b** The depletion of monocytes or B cells does not impact RTX-mediated NK elimination of CD19⁺ cells. **c** The depletion of monocytes or B cells does not impact on RTX-mediated NK cell viability. **d, e** The depletion of monocytes or B cells does not impact on RTX-mediated $CD56^{dim}$ to $CD56^{bright}$ NK cell transition. **f, g** The depletion of monocytes or B cells does not suppress RTX-mediated recovery of CD16. n=3 Cell counts in the TRA group were used to normalize cell numbers.

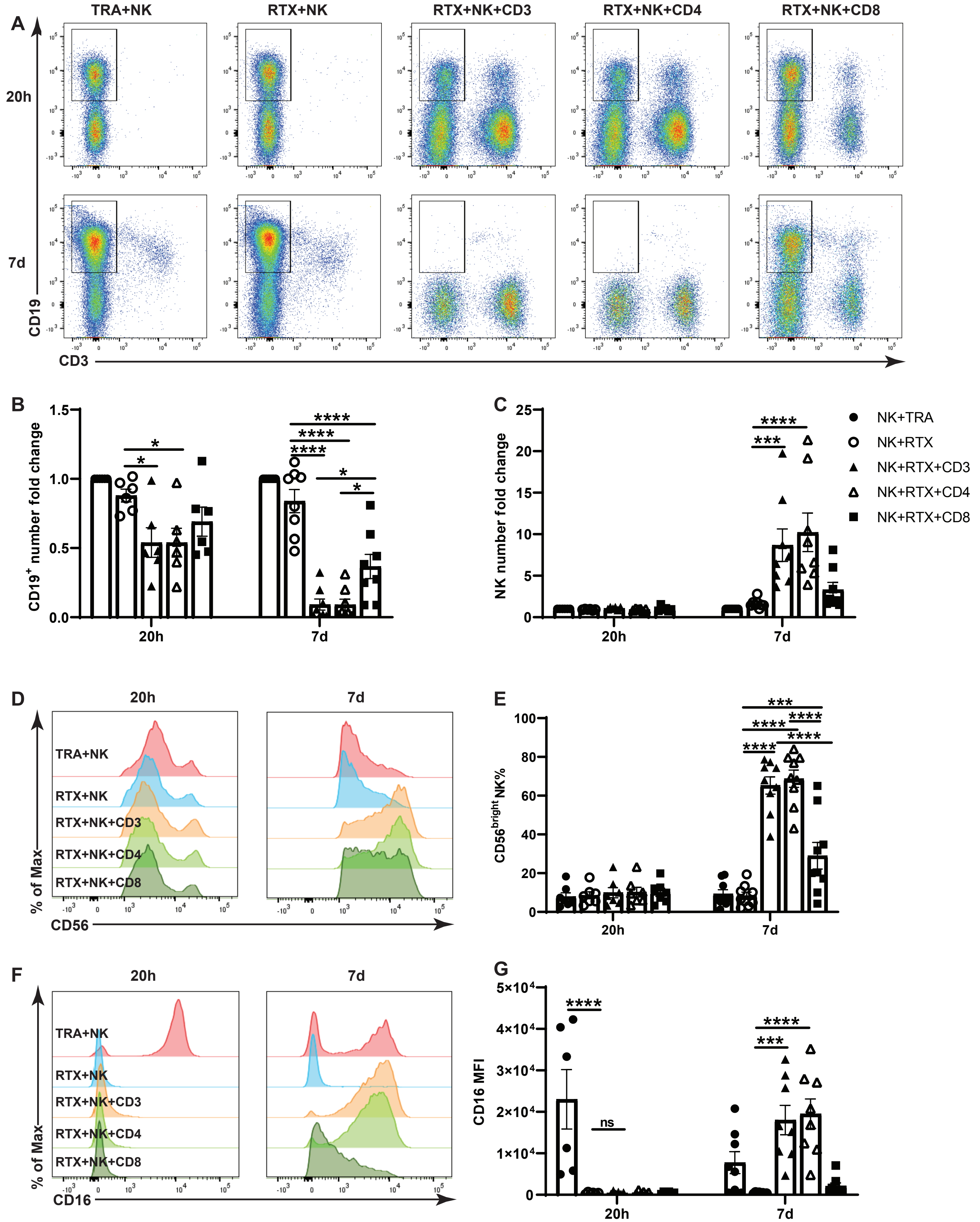

Supplementary Fig. 5 T cells, mainly CD4⁺ cells, are essential for RTX-mediated NK cell responses. Isolated NK cells were cocultured with Raji cells and RTX or TRA. T cell subsets were added to the culture based on their physiological proportion in the peripheral blood: 0.6 million $CD3^+$, 0.4 million $CD4^+$, or 0.2 million $CD8^+$ T cells. **a, b** The elimination of $CD19^+$ target cells is significantly enhanced with the presence of $CD3^+$, $CD4^+$ or $CD8^+$ T cells at day 7. $CD3^+$ and $CD4^+$ T cells improve elimination of $CD19^+$ target cells to a greater degree than $CD8^+$ T cells. **c** The number of NK cells remaining in the culture after 7 days is increased by the addition of $CD3^+$ or $CD4^+$ T cells but not by $CD8^+$ T cells. **d, e** $CD56^{dim}$ to $CD56^{bright}$ NK transition is only seen when T cells were present. $CD3^+$ and $CD4^+$ induce greater $CD56^{dim}$ to $CD56^{bright}$ NK transition than $CD8^+$ T cells. **f, g** CD16 re-expression at 7 days is only seen when $CD3^+$ or $CD4^+$ T cells are present. n=6-8. Cell counts in the TRA group were used to normalize cell numbers

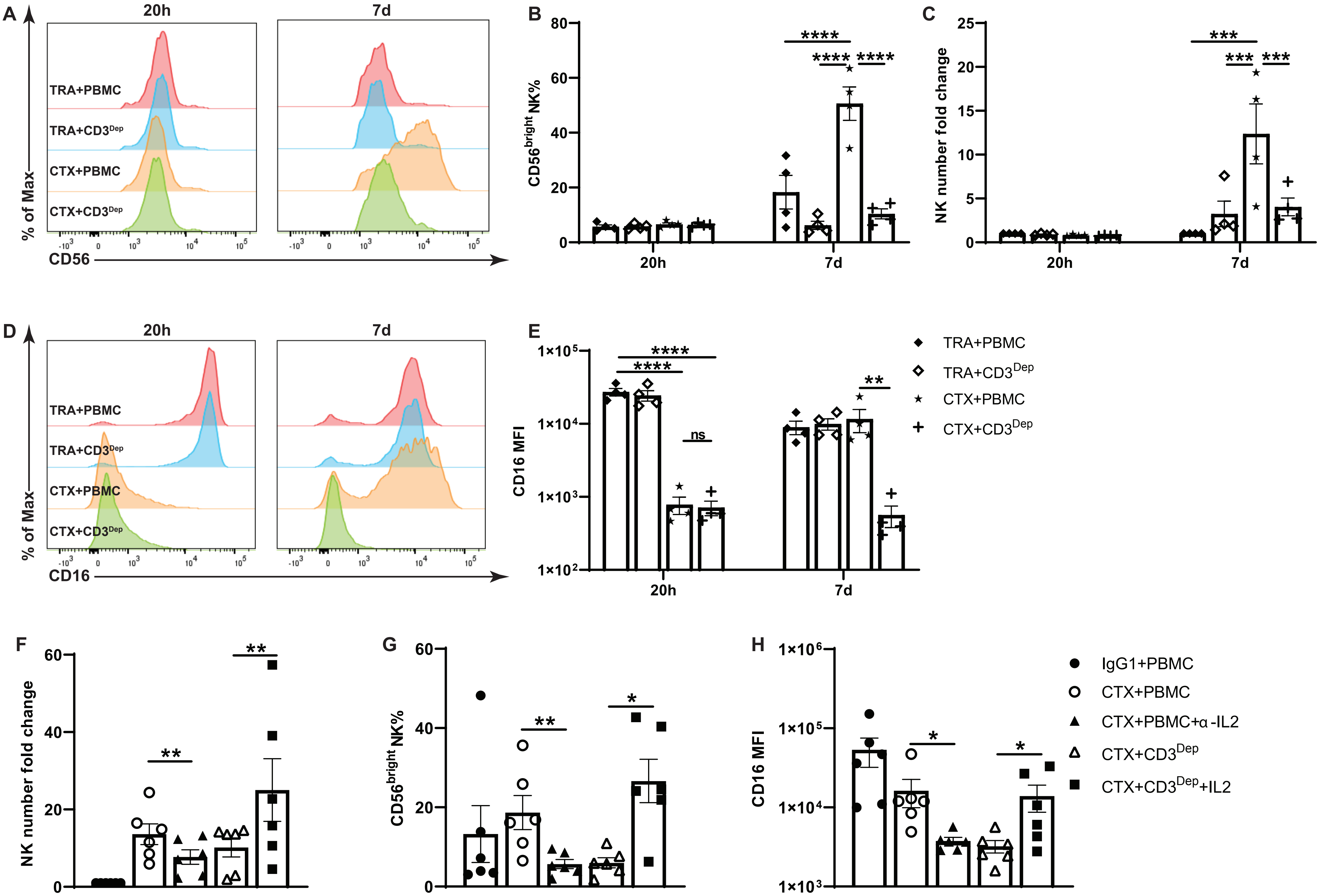

Supplementary Fig. 6 T cells maintain CTX-mediated NK cell responses via IL2. Unfractionated PBMCs or PBMCs depleted of CD3$^+$ T cells were cocultured with SQ20B cells and CTX or TRA. **a-e** On day 7, CTX induces CD56^{dim} to CD56^{bright} NK cell transition, maintains NK cell viability, and CD16 re-expression by NK cells after the initial downregulation at 20 hours in unfractionated PBMCs but not in T cell-depleted PBMCs. n=4. Unfractionated PBMCs or PBMCs depleted of CD3$^+$ T cells were cocultured with SQ20B cells and CTX or IgG1 control. α-IL2 blocking mAb (10ug/ml) or recombinant IL2 (20ng/ml) was added to the coculture for 7 days. **f-h** On day 7, CTX-mediated NK cell viability, CD56^{dim} to CD56^{bright} NK transition, and CD16 re-expression on NK cells in unfractionated PBMCs were suppressed by α-IL2, and was maintained by recombinant IL2 supplementation on NK cells in T cell-depleted PBMCs. Cell counts in the TRA+PBMC group are used to normalize cell numbers. n=6

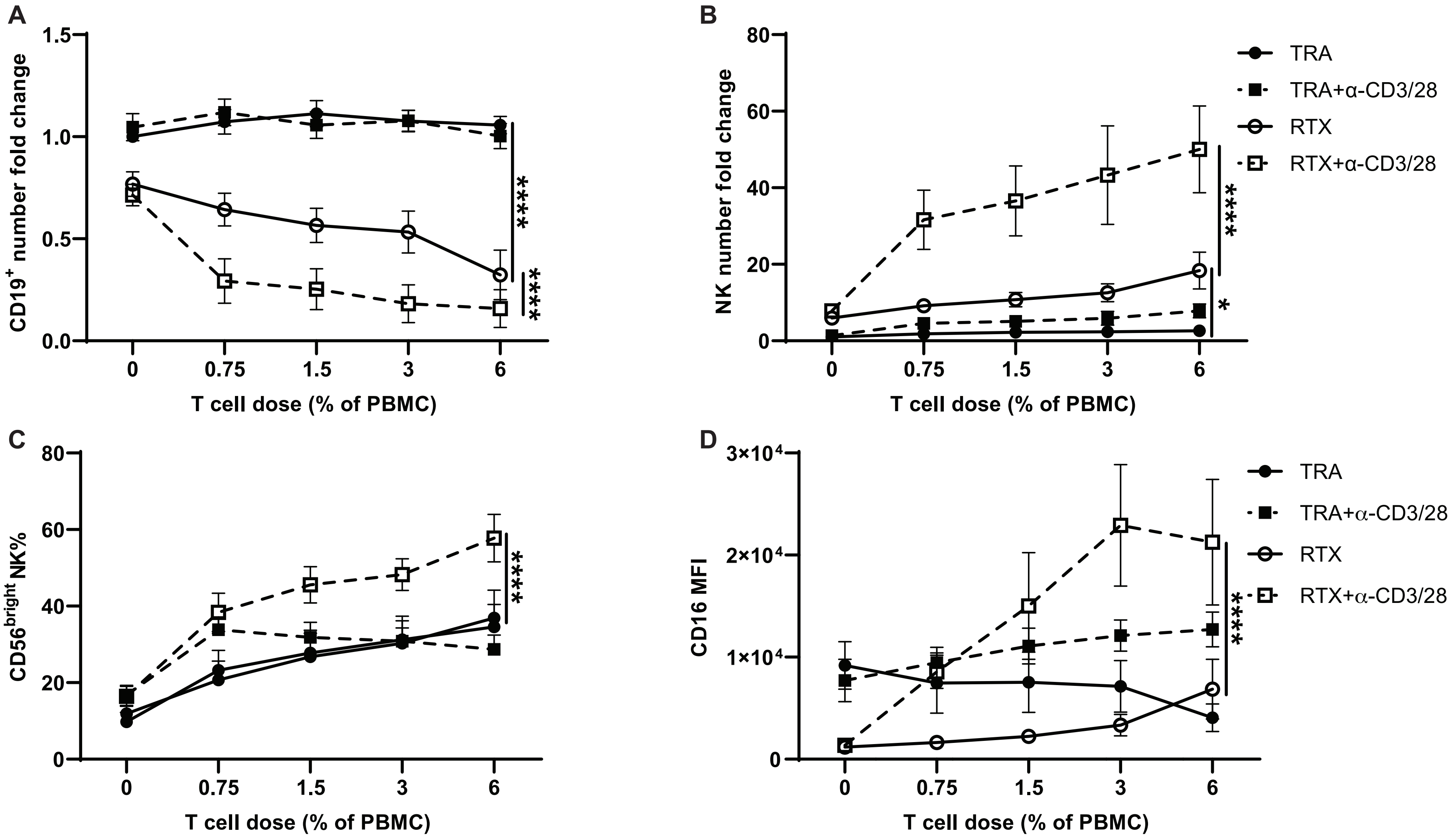

Supplementary Fig. 7 T cell activation by α-CD3/28 beads enhances RTX-mediated NK cell responses. PBMC depleted of $CD3^+$ T cells were cocultured with Raji cells and RTX or TRA for 7 days. Serial dilutions of either autologous resting or α-CD3/28-activated T cells were added to the coculture. RTX-mediated NK cell elimination of target cells (**a**), viability (**b**), $CD56^{dim}$ to $CD56^{bright}$ transition (**c**) and CD16 re-expression (**d**) is T cell dose dependent and further enhanced by T cell activation. n=7. Cell counts in the TRA group at 0% T cell dose were used to normalize cell numbers
